# The TGA Transcription Factors from Clade II Negatively Regulate the Salicylic Acid Accumulation in Arabidopsis

**DOI:** 10.3390/ijms231911631

**Published:** 2022-10-01

**Authors:** Alejandro Fonseca, Tomás Urzúa, Joanna Jelenska, Christopher Sbarbaro, Aldo Seguel, Yorley Duarte, Jean T. Greenberg, Loreto Holuigue, Francisca Blanco-Herrera, Ariel Herrera-Vásquez

**Affiliations:** 1 Millennium Science Initiative Program (ANID), Millennium Institute for Integrative Biology (iBio), Santiago 8331150, Chile; 2 Department of Plant Biology, Swedish University of Agricultural Sciences (SLU), 75007 Uppsala, Sweden; 3 Millennium Science Initiative Program (ANID), Millennium Nucleus for the Development of Super Adaptable Plants (MN-SAP), Santiago 8331150, Chile; 4 Department of Molecular Genetics and Cell Biology, The University of Chicago, Chicago, IL 60637, USA; 5 Center for Bioinformatics and Integrative Biology, Facultad de Ciencias de la Vida, Universidad Andrés Bello, Santiago 8370146, Chile; 6 Facultad de Ciencias Biológicas, Pontificia Universidad Católica de Chile, Santiago 8331150, Chile; 7 Centro de Biotecnología Vegetal, Facultad de Ciencias de la Vida, Universidad Andres Bello, Santiago 8370146, Chile; 8 Center of Applied Ecology and Sustainability (CAPES), Santiago 8320000, Chile

**Keywords:** salicylic acid, TGA transcription factors, *Pseudomonas syringae*, AvrRPM1, UV-C, *tga256*, *PR-1*, pathogenesis-related 1

## Abstract

Salicylic acid (SA) is a hormone that modulates plant defenses by inducing changes in gene expression. The mechanisms that control SA accumulation are essential for understanding the defensive process. TGA transcription factors from clade II in Arabidopsis, which include the proteins TGA2, TGA5, and TGA6, are known to be key positive mediators for the transcription of genes such as *PR-1* that are induced by SA application. However, unexpectedly, stress conditions that induce SA accumulation, such as infection with the avirulent pathogen *P. syringae* DC3000/AvrRPM1 and UV-C irradiation, result in enhanced *PR-1* induction in plants lacking the clade II TGAs (*tga256* plants). Increased *PR-1* induction was accompanied by enhanced isochorismate synthase-dependent SA production as well as the upregulation of several genes involved in the hormone’s accumulation. In response to avirulent *P. syringae*, *PR-1* was previously shown to be controlled by both SA-dependent and -independent pathways. Therefore, the enhanced induction of *PR-1* (and other defense genes) and accumulation of SA in the *tga256* mutant plants is consistent with the clade II TGA factors providing negative feedback regulation of the SA-dependent and/or -independent pathways. Together, our results indicate that the TGA transcription factors from clade II negatively control SA accumulation under stress conditions that induce the hormone production. Our study describes a mechanism involving old actors playing new roles in regulating SA homeostasis under stress.

## 1. Introduction

Salicylic acid (SA) is a plant hormone present in the whole characterized plant kingdom. It is involved in the defense response against pathogens. SA accumulates when plant cells detect a microorganism by recognizing a Pathogen-Associated Molecular Pattern (PAMP) by a surface receptor, inducing a defense response called PAMP-triggered immunity (PTI). Additionally, plants have evolved to recognize virulence effectors produced by pathogens, which are translocated into the plant cell or produced there. This recognition induces the defense response called effector-triggered immunity (ETI). Both PTI and ETI generate and require SA accumulation; thus, plants defective in the SA accumulation are deficient in the defense response against biotrophic and many hemi-biotrophic pathogens (reviewed in [1]).

SA has been associated with developmental processes and can influence the tradeoff between plant growth and defense [2,3,4]. For instance, the hyper-accumulation of the hormone, coupled with increased defense, generates dwarf phenotypes [5,6,7,8]. Additionally, exogenous SA applications can modulate root architecture through modulation of the distribution of auxin [9,10] and impact pollen tube elongation [11]. Due to the participation of SA in this delicate countervailing between defense and developmental processes, the control of production and bioavailability of the hormone may be subject to negative as well as positive regulation.

In plants, SA is produced from chorismate in plastids through two different pathways that start in chloroplasts: the phenylalanine ammonia-lyase (PAL) pathway and the isochorismate (IC) pathway. In the PAL pathway, chorismate is converted into subsequent steps to phenylalanine, trans-cinnamic acid, and benzoic acid, and then into SA (reviewed on [12]). In the IC pathway, chorismate is converted into IC by the isochorismate synthase enzyme [13,14]. The IC is then exported from chloroplasts to the cytosol by the MATE transporter EDS5 [15], and it is transformed into isochorismate-9-glutamate (IC-9-Glu) by the PBS3 protein (and possibly other GH3 family proteins) in the cytoplasm [16]. Subsequently, IC-9-Glu is spontaneously converted into SA with the contribution of the EPS1 protein [16]. The IC pathway is the primary source of SA production in response to pathogens [14,17].

TGA transcription factors are a family of proteins involved in responses to stress, nutrition, and developmental processes. The Arabidopsis genome harbors 10 genes that code for TGA members. They are classified into five clades according to their sequence similarity [18]. Clades I to III have been associated with the defense response against pathogens. TGA3 from clade III participates in the activation of SA-dependent gene expression [19], and also defense triggered by cytokinins [20]. TGA1 and TGA4 from clade I participate in the root response to nitrate [21], but also function as positive regulators of the defense response [19,22]. They do this by controlling SA accumulation through the transcriptional control of *CBP60g* and *SARD1* [23], which in turn control the expression of the *ISOCHORISMATE SYNTHASE 1* (*ICS1*) gene [24]. TGA2, TGA5, and TGA6, which comprise clade II, are essential components in the control of redox homeostasis in response to stress and detoxification in response to oxylipins and xenobiotics [25,26,27,28]. In the defense response against pathogens, the TGA proteins from clade II play an essential role in the long-lasting and broad-spectrum whole plant defense process called systemic acquired resistance (SAR) that is induced after a local infection with pathogens that are typically avirulent [29,30]. These TGA proteins are also central players in the expression of genes downstream of SA production [31].

Here we describe a new function of the TGA transcription factors belonging to clade II. They act as negative regulators of SA biosynthesis in stress conditions that induce SA accumulation in Arabidopsis plants. This discovery provides a new understanding of the regulation of SA homeostasis.

## 2. Results

### 2.1. Treatments Known to Induce SA Accumulation in Wild-Type Upregulate PR-1 in tga256 Mutant Plants

**Figure 1:**
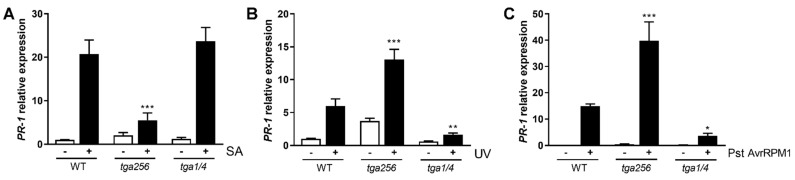
The TGA class II transcription factors negatively regulate *PR-1* expression in conditions that induce SA accumulation. *PR-1* transcript levels were evaluated in WT, *tga256* triple mutants, and *tga1*/*4* double mutants 24 h after (**A**) treatments with exogenous SA, (**B**) irradiation with UV-C, and (**C**) inoculation with avirulent *P. syringae* DC3000 (Pst) AvrRPM1. Fifteen-day-old plate-grown seedlings were used for SA and UV-C treatments, and 4-week-old soil-grown plants were used for the Pst inoculations. In (**A**), seedlings were transferred from plates to liquid ½MS media supplemented with 0.5 mM SA (+) or without the hormone (−). In (**B**), seedlings were irradiated with UV-C for 20 min (+). Plants covered with a UV-C filter were used as controls (−). After the treatments, plants were transferred to normal growth conditions. In (**C**) plants were infiltrated with Pst DC3000/AvrRPM1 OD_λ600_ = 0.01 (+) or 10 mM MgCl_2_ (−) as control. Plants were frozen after 24 h of the different treatments. RNA was extracted, cDNA was synthesized, and *PR-1* expression was evaluated by qPCR. Bars indicate the mean of the relative expression of *PR-1* ± SEM of three biological replicates. Asterisks indicate statistical differences according to a 2-way ANOVA and Dunnett’s post-test comparing the mutant genotypes with the WT plants (*: *p* < 0.05, **: *p* < 0.01, ***: *p* < 0.001). No statistically significant differences were found in control conditions (−) between WT and *tga256* mutant plants (*p* = 0.9037 in (**A**), *p* = 0.0640 in (**B**), and *p* = 0.9951 in (**C**)).

In Arabidopsis, the expression of the defense gene *PR-1* depends on members of the TGA transcription factors’ family [19]. In the *tga256* triple mutant plant lacking the TGA clade II, *PR-1* expression is highly reduced in response to SA treatments compared to WT plants (Figure 1a). This result supports the dependency of TGA transcription factors from clade II for the induction of *PR-1* in response to isonicotinic acid (INA, a functional analog of SA) treatment, as previously reported by [29]. In contrast, the tga256 mutant showed higher levels of *PR-1* than were seen in WT in response to UV-C or P. syringae pv tomato (Pst) DC3000/AvrRPM1 (Figure 1b,c). This was surprising since both treatments are known to induce SA accumulation in WT [32,33]. The *tga2-1*/*tga5-1* double mutant did not show statistically significant differences in *PR-1* expression compared to the control genotype (Figure S1). We also tested *PR-1* expression in plants lacking TGA transcription factors from clade I (TGA1 and TGA4), described as positive regulators of SA accumulation [23]. These plants showed *PR-1* expression that was lower than that seen in WT plants in response to either UV-C treatment or Pst DC3000/AvrRPM1 inoculation (Figure 1b,c).

**Figure 2:**
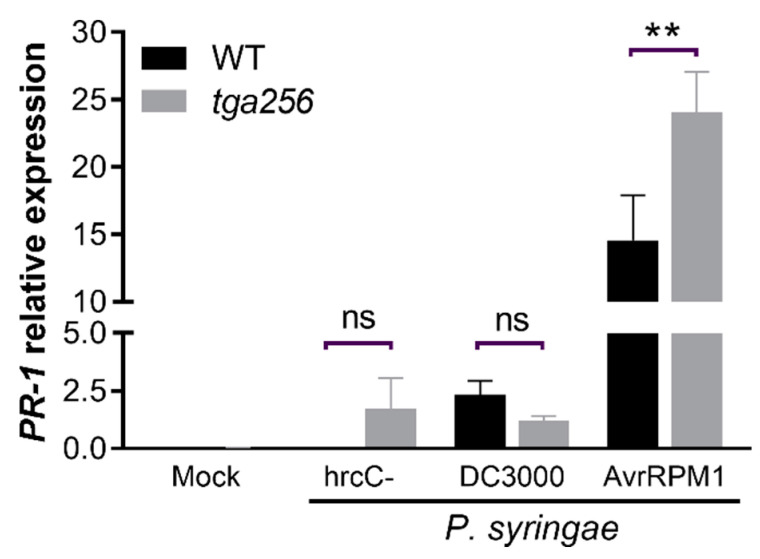
The TGA class II regulation of *PR-1* expression is active during the ETI response triggered by Pst DC3000/AvrRPM1, but not during PTI or ETS responses. Four-week-old WT and *tga256* mutant plants were syringe-infiltrated with a virulent *Pseudomonas syringae* DC3000 strain (DC3000), a non-pathogenic hrcC mutant of Pst DC3000 strain (hrcC^−^), or the Pst DC3000 avirulent strain carrying the AvrRPM1 gene (AvrRPM1). All strains were infiltrated at OD_λ600_ = 0.01 in a 10 mM MgCl_2_ solution. In total, 10 mM MgCl_2_ was used as a control (mock). Twenty-four hours post inoculation (hpi), the RNA was extracted, and *PR-1* expression was evaluated. Bars represent the mean of *PR-1* relative expression ± SEM of four biological replicates. Asterisks indicate statistical differences compared to WT plants according to a 2-way ANOVA and Sidak’s post-test (**: *p* < 0.01), ns = not significant.

To further explore the roles of the clade II TGA factors, we evaluated *PR-1* expression in the *tga256* mutant plants during the PTI and effector-triggered susceptibility (ETS) triggered by a *Pseudomonas* strain able to produce disease in the host. The PTI response was induced by the *P. syringae* pv *tomato* DC3000 hrcC^−^ strain that does not secrete virulence effectors [34], while the ETS response was triggered by the virulent *Pseudomonas* strain Pst DC3000 [35]. We measured *PR-1* transcript 24 h after inoculation, and we found that Pst DC3000 and Pst DC3000/AvrRPM1 strains induced the *PR-1* transcript accumulation compared to the mock condition on WT plants (*p* = 0.0093 and *p* = 0.0045, respectively). Nevertheless, we only found a statistically significant difference between WT and *tga256* plants in the response (ETI) triggered by Pst DC3000/AvrRPM1 (Figure 2). Even though we observed a slight difference in the *PR-1* transcript in the response triggered by Pst hrcC^−^ between the analyzed genotypes, it was not statistically significant (*p* = 0.297, Figure 2).

These results show multiple roles for clade II transcription factors: they are needed for the full *PR-1* response to exogenous SA, but they somewhat suppress the *PR-1* response to UV-C or, specifically, the ETI-inducing Pst DC3000/AvrRPM1 strain, as indicated by the increased expression of *PR-1* in the triple mutant *tga256* plants.

### 2.2. TGA2 Negatively Regulates the Expression of Isochorismate Pathway-Related Genes upon Stress

**Figure 3:**
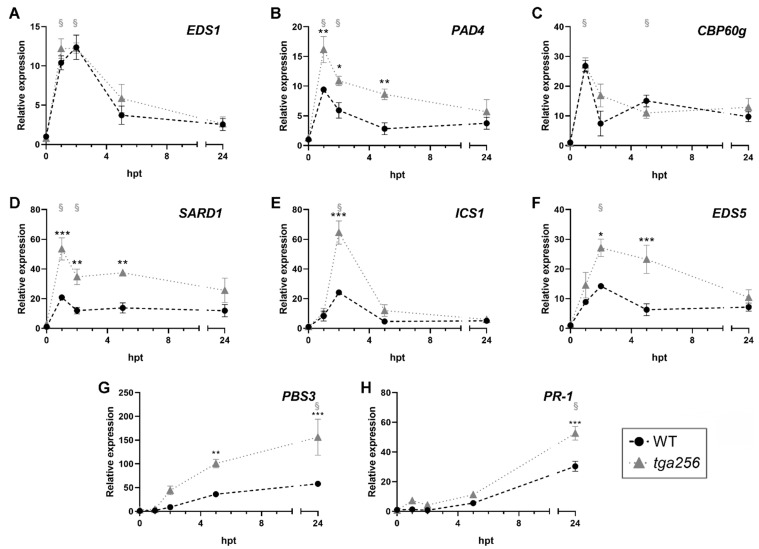
UV-C treatment upregulates the expression of genes that participate in SA accumulation in the *tga256* triple mutant plants. Fifteen-day-old WT (black circles) and *tga256* mutant (gray triangles) seedlings were treated with UV-C radiation for 20 min, and then were transferred to normal growth conditions. Samples were collected 1, 2, 5, and 24 h post treatment (hpt). Non-irradiated plants were used as controls (0 hpt). RNA was extracted and the gene expression of (**A**) *EDS1*, (**B**) *PAD4*, (**C**) *CBP60g*, (**D**) *SARD1*, (**E**) *ICS1*, (**F**) *EDS5*, (**G**) *PBS3*, and (**H**) *PR-1* genes was evaluated by RT-qPCR. The data represent the mean of relative gene expression ± SEM from three biological replicates. Asterisks indicate statistical differences according to a 2-way ANOVA and Sidak’s post-test comparing the *tga256* mutants with the WT plants at a given time point (*: *p* < 0.05, **: *p* < 0.01, ***: *p* < 0.001). § indicates statistically significant differences in the gene expression at the indicated time compared to the control condition (0) in the WT genotype (*p* < 0.05, Dunnet’s post-test).

**Figure 4:**
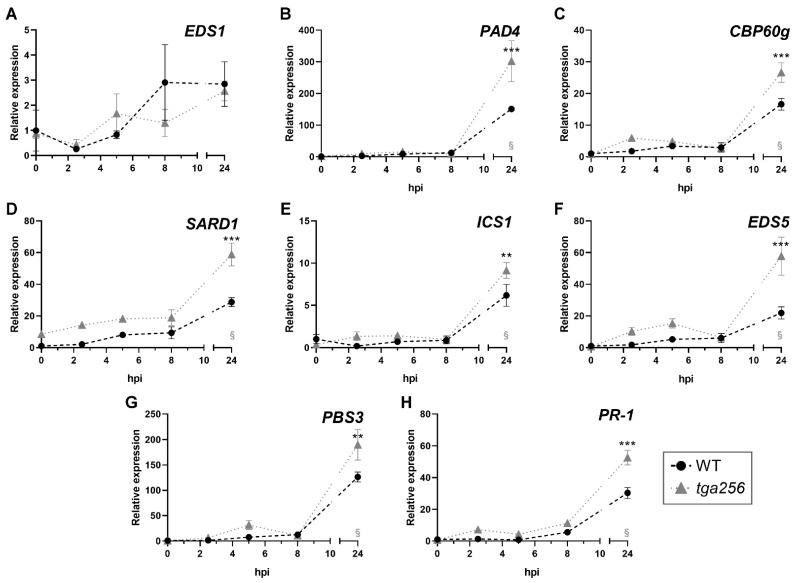
Inoculation with the avirulent *P. syringae* DC3000/AvrRPM1 induces the upregulation of genes that participate in SA accumulation in the *tga256* mutant compared to the WT plants. Four-week-old WT (black circles) and *tga256* mutant (gray triangles) plants were syringe-infiltrated with Pst DC3000/AvrRPM1 OD_λ600_ = 0.01. Samples were collected 2.5, 5, 8, and 24 h post inoculation (hpi). Non-inoculated plants were used as controls. RNA was extracted and the gene expression of (**A**) *EDS1*, (**B**) *PAD4*, (**C**) *CBP60g*, (**D**) *SARD1*, (**E**) *ICS1*, (**F**) *EDS5*, (**G**) *PBS3*, and (**H**) *PR-1* genes was evaluated by RT-qPCR. The data represents the mean of the relative gene expression ± SEM from four biological replicates. Asterisks indicate statistical differences according to a 2-way ANOVA and Sidak’s post-test comparing the *tga256* mutants with the WT plants at a given time point (**: *p* < 0.01, ***: *p* < 0.001). § indicates statistically significant differences in the gene expression at the indicated time compared to the control condition (0) in the WT genotype (*p* < 0.05, Dunnet’s post-test).

As *PR-1* induction is often associated with SA accumulation [36], we evaluated the possible involvement of the TGA factors from clade II on the SA biosynthetic pathway. To assess this, we evaluated the expression of biosynthetic and regulatory genes involved in the IC pathway in plants irradiated with UV-C (Figure 3) and in the ETI response triggered by Pst DC3000/AvrRPM1 (Figure 4). The genes *ENHANCED DISEASE SUSCEPTIBILITY-1* (*EDS1*) and *PHYTOALEXIN DEFICIENT-4* (*PAD4*), which constitute a crucial regulatory node in the defense response [37], were both significantly induced 1 h after UV-C treatment in WT plants relative to the control condition (Figure 3a,b). In the *tga256* mutant plants, the induction of EDS1 was similar to WT plants (Figure 3a), whereas the *PAD4* gene was significantly increased at 1, 2, and 5 h post treatment (hpt, Figure 2b). Expression of the transcription factors *SYSTEMIC ACQUIRED DEFENSE DEFICIENT-1* (*SARD1*) and *CALMODULIN BINDING PROTEIN-60g* (*CBP60g*), which transcriptionally control the expression of the *ISOCHORISMATE SYNTHASE-1* (*ICS1*) gene [24], showed a maximal induction one hour after the UV-C treatment in WT plants (Figure 2c,d). In the *tga256* mutant plant, the expression of *SARD1* was significantly increased compared to WT plants at multiple early time points (Figure 3d). The expression of *CBP60g* was not affected in the *tga256* mutant compared to WT plants (Figure 3c). *ICS1* maximal induction was reached 2 h after the UV-C treatment (Figure 3e). This is consistent with the induction kinetics of *SARD1* and *CBP60g*, which show peaks 1 h before that of the *ICS1* transcript (Figure 3c,d). *ICS1* expression was significantly increased in the *tga256* mutant compared to WT plants at 2 hpt, with an approximately 2.9-fold increase. The expression of the IC transporter *ENHANCED DISEASE SUSCEPTIBILITY-5* (*EDS5*) [15] showed a maximal induction 2 hpt in WT plants (Figure 3f). This induction was significantly increased in the *tga256* mutant (Figure 3f). The *PBS3* gene that participates in the conversion from isochorismate to SA [16], was hyper-induced in the *tga256* mutants compared to the WT plants at 5 and 24 hpt (Figure 3g). Consistent with the results shown in Figure 1b, the expression of *PR-1* was increased in the *tga256* compared to WT plants at 24 hpt (Figure 3h). In summary, the transcripts of the *PAD4*, *SARD1*, *ICS1*, *EDS5*, *PBS3*, and *PR-1* genes showed an increased expression in the *tga256* mutant plants compared to the WT plants under UV-C treatment.

We evaluated expression of the same suite of genes in plants challenged with Pst DC3000/AvrRPM1 to induce the ETI response. The treatment increased the transcript level of the genes *PAD4*, *SARD1*, *CBP60g*, *EDS5*, *ICS1*, and *PSB3* in WT plants after 24 h post infection (hpi, *p* < 0.05, Figure 4). Although *EDS1* gene seemed to be upregulated in response to the infection after 24 hpi, statistical analysis showed no significant differences compared with the control condition (Figure 4a, *p* > 0.05, 2-way ANOVA). The comparison of gene expression between genotypes showed that the *tga256* mutant plants displayed an increased transcript accumulation at 24 hpi in all genes induced by the treatment (Figure 4b–h). No differences were detected in the expression of *EDS1* (Figure 4a).

**Figure 5:**
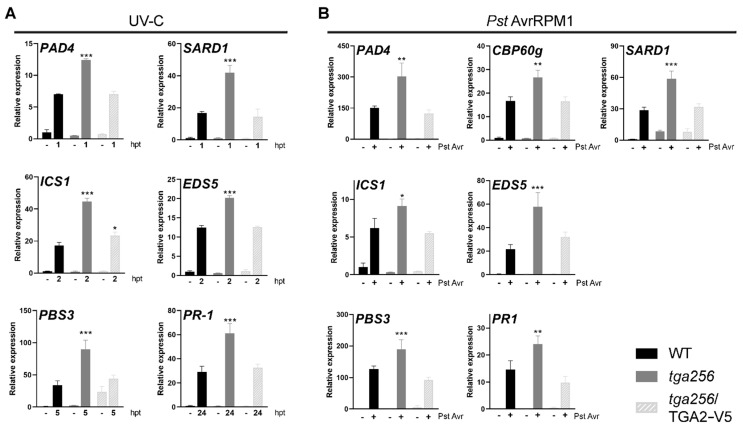
The expression of TGA2 in the *tga256* mutant background rescues the upregulated expression of genes associated with SA accumulation upon UV-C and Pst DC3000/AvrRPM1 treatments. WT, *tga256*, and *tga256*/TGA2-V5 plants were (**A**) irradiated with UV-C or (**B**) inoculated with Pst DC3000/AvrRPM1 (Pst Avr). Unirradiated or non-inoculated plants were used as controls (−), respectively. The expression of the indicated genes was evaluated at the time when the genes were differentially expressed between the genotypes WT and *tga256*. In the case of pathogen inoculation, gene expression was measured at 24 hpi (+). Samples were frozen and RNA was extracted. Gene expression was evaluated by RT-qPCR. The data represents the mean of the relative gene expression ± SEM. Asterisks indicate statistical differences between the mutant genotypes compared to the WT plants according to a 2-way ANOVA and Sidak’s post-test (*: *p* < 0.05, **: *p* < 0.01, ***: *p* < 0.001). Three biological replicates were evaluated for UV-C treatments; four biological replicates were evaluated in the Pst DC3000/AvrRPM1 treatments.

To determine whether the altered expression profiles observed in the *tga256* mutant were due to the lack of function of the TGA clade II, we used a rescued transgenic line that expresses V5 epitope-tagged TGA2 (TGA2-V5) controlled by a constitutive promoter (*tga256*/TGA2-V5 [28]). We evaluated the genes involved in SA biosynthesis that were differentially regulated in the *tga256* mutant plants in response to the treatments (Figure 5). We made measurements at times in which WT and *tga256* genotypes displayed statistically significant differences in the UV-C and Pst DC3000/AvrRPM1 treatments (from Figure 4 and Figure 5). The expression of *PAD4*, *SARD1*, *EDS5*, *PBS3*, and *PR-1* genes was fully restored to WT levels in the *tga256*/TGA2-V5 complemented line in UV-C treatment (Figure 5a). TGA2-V5 expression in the *tga256* background partially rescued the gene expression of *ICS1* (*p* = 0.0257 comparing WT and *tga256*/TGA2-V5, and *p* < 0.0001 comparing *tga256* and the rescued line according to a 2-way ANOVA and Sidak’s post-test). Additionally, the *tga256*/TGA2-V5 rescued line showed restored expression of all tested genes to levels similar to WT plants in the inoculation with Pst DC3000/AvrRPM1 (Figure 5b). These experiments show that TGA2 acts as a negative regulator of the expression of genes involved in SA signaling as well as SA accumulation in response to UV-C treatments and ETI triggered by Pst DC3000/AvrRPM1.

### 2.3. TGA2 Controls the Accumulation of ICS1 and SA under Stress

In Arabidopsis, the isochorismate (IC) pathway is responsible for 90% of the SA produced after UV-C irradiation [38] and in the response against pathogens [14]. The first and rate-limiting step in the IC pathway is the conversion of chorismate to isochorismate, catalyzed by the ICS1 enzyme. To test the role of the clade II TGA factors in impacting the IC pathway, we evaluated ICS1 accumulation after UV-C treatment and Pst DC3000/AvrRPM1 inoculation using an antibody that specifically recognizes the ICS1 protein [39].

**Figure 6:**
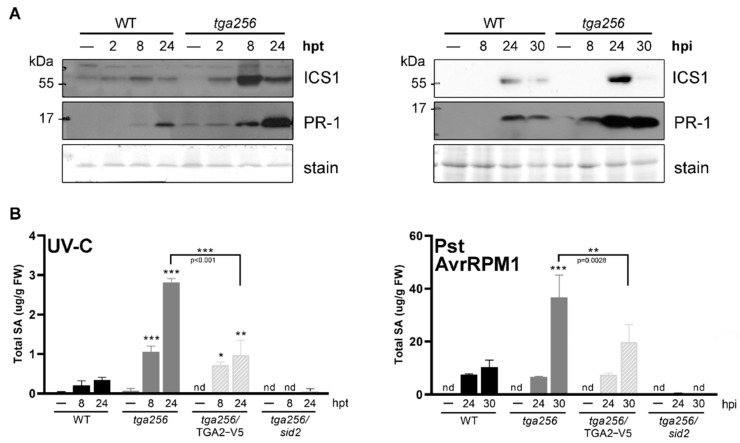
TGA class II negatively regulates the SA accumulation in Arabidopsis plants under stress. (**A**) Fifteen-day-old WT and *tga256* mutant plants were irradiated with UV-C as described in methods (left panel). Additionally, 4-week-old WT and *tga256* mutant plants were inoculated with Pst DC3000/AvrRPM1 OD_λ600_ = 0.01 (right panel). Plants were frozen at indicated time points. ICS1 and *PR-1* protein abundance was evaluated by Western blot. In the top right blot (ICS1), chemiluminescence was detected with CCD camera and all other blot signals were detected by X-ray film. Similar results were obtained in two additional experiments with UV-C and one additional experiment with Pst inoculation. (**B**) SA accumulation was measured in WT, *tga2-1*/*tga5-1*/*tga6-1* (*tga256*), *tga256*/TGA2-V5 and *tga2-1*/*tga5-1*/*tga6-1*/*sid2-2* (*tga256*/*sid2*) plants. Unirradiated and non-inoculated plants were used as control (−). Bars represent the mean of SA detected by HPLC relative to the fresh weight of the sample ± SEM (mg SA/g FW, three biological replicates). Asterisks indicate statistically significant differences between the mutant genotypes and the WT plants at a given time point according to a 2-way ANOVA analysis and Sidak’s post-test (*: *p* < 0.05, **: *p* < 0.01, ***: *p* < 0.001). nd indicates that SA was not detected in the samples.

WT plants showed a modest increase in ICS1 protein at 8 h after the UV-C treatment, whereas the *tga256* mutants showed a higher accumulation of ICS1 upon stress. This difference reached its maximal effect 8 h after the UV-C treatment (Figure 6a, left panel). Consistent with the transcript evaluation (Figure 1b), the accumulation of the *PR-1* protein was increased in the *tga256* mutant plants compared to the WT starting at 8 hpt, reaching its maximal accumulation at 24 hpt (Figure 6a, left panel).

We detected the ICS1 protein at 24 hpi with the Pst DC3000/AvrRPM1 strain in WT plants. Consistent with the result in response to UV-C, the *tga256* mutant plants displayed increased ICS1 accumulation compared to WT plants at 24 hpi (Figure 6a, right panel). *PR-1* protein levels were also higher in the *tga256* mutant plants compared to the WT in the ETI response triggered by Pst DC3000/AvrRPM1 at 24 and 30 hpi. The dynamics of ICS1 and *PR-1* protein accumulation correlated very well with transcript induction patterns (Figure 3 and Figure 4) and were slower during Pst DC3000/AvrRPM1 infection compared to UV-C treatment.

To test whether higher ICS1 was associated with higher SA accumulation, we evaluated SA accumulation in the plants treated with UV-C and inoculated with Pst DC3000/AvrRPM1. In both treatments, we observed an accumulation of the hormone in WT plants during the evaluated time-course (8 and 24 after in UV-C and 24 and 30 after Pst DC3000/AvrRPM1 inoculation). SA accumulation was higher in *tga256* plants compared to the WT. Production of the TGA2-V5 protein in the *tga256* background (*tga256*/TGA2-V5) reversed the phenotype found in the *tga256* mutant plant in both UV-C and Pst DC3000/AvrRPM1 treatments. These experiments indicate that TGA2 acts as a negative regulator of SA accumulation through the activation of the IC pathway.

In order to evaluate whether the hyper-accumulation of SA in the *tga256* mutant plant requires the IC pathway, we generated the *tga2-1*/*tga5-1*/*tga6-1*/*sid2-2* (*tga256*/*sid2*) mutant plant by crossing the *tga256* mutant with the *sid2-2* mutant that lacks the ICS1 gene (Figure S2). The quadruple mutant did not show the SA increase displayed in the *tga256* mutant plant after the UV-C treatment or Pst DC3000/AvrRPM1. This indicates that the IC pathway is the primary source of the SA hyper-accumulation in the *tga256* mutant plant.

### 2.4. NPR1 Does Not Affect the Expression of IC Genes during Stress

**Figure 7:**
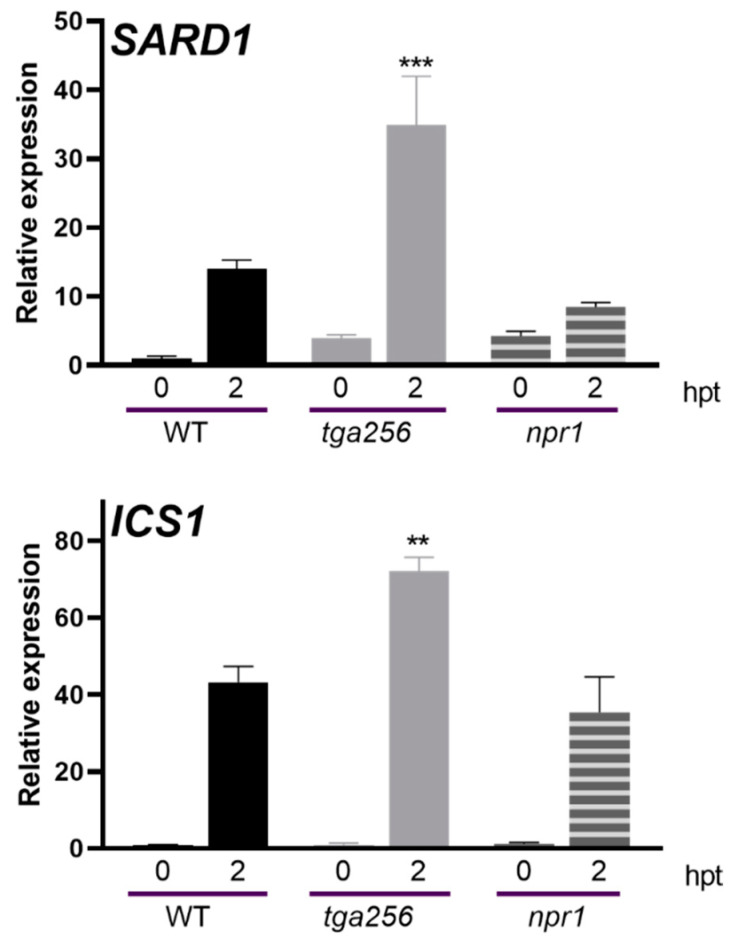
NPR1 does not participate in the regulation of genes related to SA accumulation under stress. Fifteen-day-old WT, *tga2-1*/*tga5-1*/*tga6-1* (*tga256*), and *npr1-1* (*npr1*) mutant seedlings were treated with UV-C. Non-irradiated seedlings were used as control (0). Treated seedlings were frozen at 2 h post treatment (hpt). RNA was extracted, and the *SARD1* and *ICS1* gene expression was evaluated by RT-qPCR. Bars represent the mean of the relative expression ± SEM from four biological replicates. Asterisks indicate statistically significant differences between the mutant genotypes and the WT plants according to a 2-way ANOVA analysis and Sidak’s post-test (**: *p* < 0.01, ***: *p* < 0.001).

Together with the TGA transcription factors, NPR1 co-regulates gene expression downstream of SA accumulation and is one of the SA receptors [40,41,42]. To evaluate the participation of NPR1 in the repression of the IC pathway during stress, we measured the *SARD1* and *ICS1* transcript levels in *npr1* mutant plants. Unlike the *tga256* plants, the transcripts of these genes were not upregulated in the *npr1* mutant compared to the WT plants in response to UV-C (Figure 7). Additionally, we evaluated the *PR-1* transcript, whose expression is activated by the NPR1 protein in response to SA [43]. As expected, we observed that the *PR-1* expression was not activated in the *npr1* mutant compared to the WT plants upon UV-C irradiation (Figure S3).

## 3. Discussion

The transcription factors belonging to clade II of the TGA family in Arabidopsis were previously described as positive regulators of the transcriptional response to an SA analog [29]. Here we described the participation of this clade of TGA genes as negative regulators of SA accumulation in response to both an abiotic and a biotic stress: UV-C and the ETI triggered by Pst DC3000/AvrRPM1, respectively.

*PR-1* is a well-known SA-responsive gene [36]. TGA2 directs and positively regulates *PR-1* expression in response to the SA analog isonicotinic acid (INA) [29]. As well, the clade II proteins are needed for *PR-1* induction in response to SA (this work). It was initially surprising that *tga256* plants subjected to stresses that induce SA were still able to induce *PR-1* and had even higher expression than in WT. This result suggests that TGA2 is not required for *PR-1* activation in response to stress, unlike previously described for SA analog treatments [29]. However, Tsuda et al. [44] showed that *PR-1* is subject to both SA-dependent and SA-independent regulation during ETI responses triggered by Pst DC3000/AvrRPM1 and Pst DC3000/AvrRPT2. They demonstrated that SA-independent upregulation is mediated by the activation of Mitogen-Activated Protein Kinase 3 and 6 (MPK3/6) in response to Pst DC3000/AvrRPT2 [44]. MPK3/6 activation is also triggered in response to Pst D3000/AvrRPM1 [45]. *PR-1* activation in these conditions could be mediated by transcription factors induced by the ETI response in an SA-independent fashion. This is the case for WRKY50, described as a *PR-1* positive regulator that binds to its promoter to control the expression in the absence of the TGA factors belonging to clade II [46].

**Figure 8:**
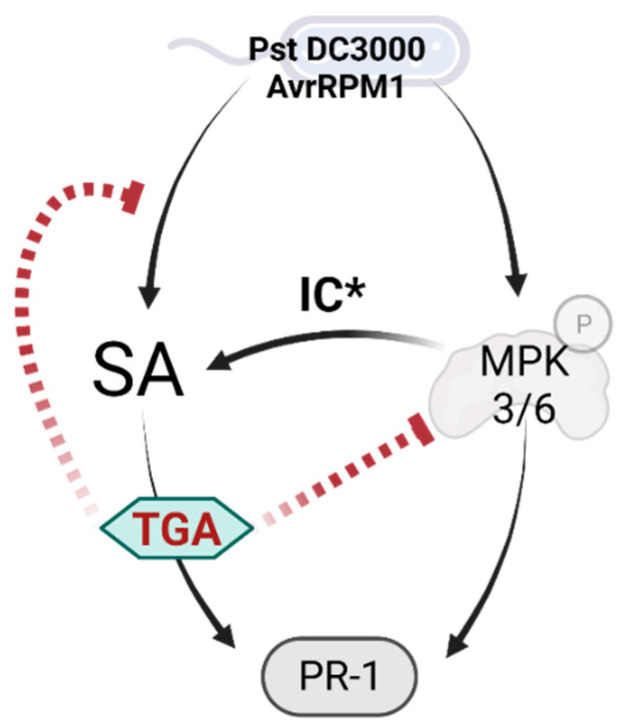
The ETI response activates *PR-1* in an SA-dependent and an SA-independent fashion. The activation of *PR-1* depends on TGA from clade II in the pathway dependent on SA. On the other hand, Mitogen–Activated Protein Kinases 3 and 6 (MPK3/6) activation triggers the SA-independent *PR-1* induction [44]. Additionally, the activation of MPK3 induces the activation of genes that participate in SA accumulation (IC*) independently of SA [44]. According to our model, TGA factors from clade II (TGA) can repress the MPK3/6 branch. Thus, in the *tga256* mutant plants, the repression of TGA over the MPK branch is absent, permitting increased SA accumulation (Figure 6), the genes (Figure 4), and the *PR-1* expression (Figure 1) dependent on MPKs. It is also possible, and not mutually exclusive, that upon stimulation of SA accumulation by ETI, the TGA factors negatively feedback on SA accumulation by suppressing a step upstream of SA accumulation, independent of MPK3/6 signaling.

The SA-independent gene induction during ETI is caused by the activation of MPK3/6 and includes the genes *SARD1*, *CBP60g*, *EDS5*, and *PBS3* [44] that together can participate in promoting SA accumulation. Indeed, Lang et al. [45] showed that constitutive activation of MPK3 produces an enhanced defense against Pst DC3000, and a dwarf phenotype that depends on SA biosynthesis [45,47]. This suggests that strong MPK3 activation induces SA accumulation. According to these data, the increase in the genes that participate in the SA accumulation in the *tga256* mutant (Figure 4), could be indicative of a negative regulation of TGA factors from clade II over the defense branch controlled by MPK3/6 in response to ETI triggered by Pst DC3000/AvrRPM1. This negative regulation could explain the *PR-1* hyper-accumulation in the *tga256* triple mutant in conditions that induce the SA accumulation (Figure 1). It is also possible that the TGA factors negatively regulate SA accumulation independent of (and/or in addition to) an effect on MPK activation. Figure 8 summarizes these possibilities.

Previously, a different clade of TGA transcription factors has been reported as regulators of the SA accumulation. Sun et al. described that TGA1 and TGA4 (from clade I) stimulate SA accumulation during PAMP-induced pathogen resistance and systemic acquired resistance [23]. TGA1 and TGA4 are required for fully activating both *SARD1* and *CBP60g* expression, which in turn transcriptionally regulate *ICS1* expression [24] and consequently SA accumulation. The *SARD1* promoter possesses two putative TGA-binding elements in the proximal region. These TGACG boxes are located at −258 and −282 nucleotides from the translation start codon. As the TGA transcription factors recognize similar cis-elements, we hypothesize the TGA transcription factors from clade II share or compete for these putative binding elements with TGA from clade I to regulate the *SARD1* expression and, consequently, the SA accumulation. Unlike *TGA1*, *TGA2* expression does not change in response to Pst DC3000/AvrRPM1 (Figure S4), suggesting the involvement of co-regulators of TGA2 that could repress genes associated with SA accumulation. NPR1 is described as the main co-regulator of the SA-mediated response [40,41]. It interacts in the nucleus with TGA2, increasing the DNA binding affinity of TGA to *PR-1* promoter [43,48]. We did not detect statistically significant changes in the expression of *SARD1* and *ICS1* genes in the *npr1* mutant plants compared to the WT (Figure 7), suggesting that NPR1 does not participate in the suppression of SA biosynthesis. This result is consistent with the hypothesis described by Rochon et al. [49], which indicates that TGA2 becomes a transcriptional activator when it interacts with transcriptional co-regulators such as NPR1 or SCL14 [50], but basally has a repressive function. Nevertheless, we cannot discard the possibility of other transcriptional co-regulators’ participation in the response, for instance, transcription factors described as TGA2-interacting proteins such as WRKY50 or TGA3, which also participate in *PR-1* regulation [46].

The TGA transcription factors from clade II have a redundant function in the process involved in the survival in SA germination and SAR [29], response to oxylipins [26], and antioxidant response to UV-B [28]. *PR-1* accumulation did not show differences between the WT and *tga25* double mutant plants (Figure S1), suggesting that TGA2 and TGA5 are redundant with TGA6 in suppressing SA accumulation. This is supported by the experiments involving the *tga256*/TGA2-V5 transgenic plant, which indicate that the expression of TGA2 alone in the *tga256* mutant background restores the gene expression and SA accumulation to WT levels (Figure 5 and Figure 8b).

According to our results, SA accumulation is negatively controlled by the TGA transcription factors from clade II in response to stresses that trigger the SA accumulation. This mechanism is a new step in the regulation of this hormone, which is fine-tune regulated due to the tradeoff between defense and developmental processes [4]. Interestingly, we detected the SA suppression by TGA class II in conditions in which the SA pathway is induced (after UV-C treatment and triggered ETI), and not in basal conditions, suggesting a feedback regulation after SA induction. Other transcription factors have been described as repressors of SA accumulation; ETHYLENE INSENSITIVE3 (EIN3) and ETHYLENE INSENSITIVE3-LIKE1 (EIL1) negatively regulate hormone accumulation by repressing the *ICS1* promoter in activated and basal conditions [51]. Accordingly, we hypothesize that the suppression by TGA class II factors could act as a braking mechanism on SA accumulation, that together with biochemical modification of SA by conjugation with other molecules (reviewed in [52]), could contribute to the homeostasis of the hormone to alleviate the detrimental effects triggered by its elevated concentrations [5,6,7,8,53,54,55].

## 4. Materials and Methods

### 4.1. Plant Growth Conditions and Treatments

*Arabidopsis thaliana* wild-type (WT) *npr1-1* [56], *tga1-1*/*tga4-1* [19], *tga2-1*/*tga5-1*/*tga6-1*, *tga2-1*/*tga5-1* [29], and *tga256*/TGA2-V5 [28], *sid2-2* [14], and *tga2-1*/*tga5-1*/*tga6-1*/*sid2-2* (this report) plants were in Columbia (Col-0) background. For UV-C and SA treatments, 14-day-old seedlings were grown in vitro in ½ MS medium supplemented with 10 g/L sucrose and 2.6 g/L Phytagel (Sigma, St. Louis, MO, USA) under controlled conditions (16 h light, 80 µmoles m^−2^ s^−1^, 22 ± 2 °C). UV-C treatments: plants were exposed for 20 min in a chamber equipped with two UV-C fluorescent tubes of 8 Watts each (λ = 254 nm) at a distance of 30 cm above the plates. UV-treated plants were subsequently placed in a growth chamber under controlled conditions for the indicated time periods. SA treatments: 14-day-old seedlings were transferred from plates to liquid ½ MS medium supplemented with 0.5 mM SA (treatment) or ½ MS alone as control. Seedling roots were in contact with the liquid media. Samples were incubated for the indicated periods of time under continuous light (80 µmoles m^−2^ s^−1^). For gene expression assays, whole seedlings were immediately frozen in liquid nitrogen and stored at −80 °C until RNA isolation. For infection assays, 4-week-old plants were grown in a soil mixture containing turba soil–perlite–vermiculite (1:1:1) under standard conditions (12 h light/12 h dark, 80 µmoles m^−2^ s^−1^, 22 ± 2 °C).

### 4.2. Bacterial Strains and Plant Infections

*Pseudomonas syringae* pv *tomato* DC3000, the isogenic avirulent strain expressing the AvrRPM1 gene (Pst DC3000/AvrRPM1) [57] or the hrcC mutated strain (Pst DC3000 hrcC^−^) [58] were grown on King B media supplemented with Rifampicin (50 μg/mL) and Kanamycin (50 mg/mL). The strains were grown at 28 °C for 24 h, centrifuged, and resuspended in 10 mM MgCl_2_. The bacteria were inoculated by syringe infiltration on the abaxial side of the leaves (OD_λ600nm_ = 0.01). Ten mM MgCl_2_ was used as a mock control.

### 4.3. Gene Expression Analysis

Total RNA was obtained from frozen samples using the TRIzol^®^ Reagent (Invitrogen, Waltham, MA, USA) according to the manufacturer’s instructions. cDNA was synthesized from each sample (1 µg of total RNA) with M-MULV reverse transcriptase (New England Biolabs, Ipswich, MA, USA) according to manufacturer’s indications. qPCR was performed with the AriaMX 3000 Real-time PCR system (Agilent, Santa Clara, CA, USA) using the BRILLIANT III ULTRA-FAST SYBR GREEN reagent (Stratagene, San Diego, CA, USA). The expression levels of the different genes were calculated relative to the *YLS8* (AT5G08290) gene. Three (Figure 1, Figure 3, Figure 5a and Figure 6) or four (Figure 2, Figure 4, Figure 5b and Figure 7) biological replicates were used in each experiment. Primers used for each gene are listed in Supplementary Table S1.

### 4.4. Protein Extracts and Immunoblot

Total protein extracts were obtained from plants by homogenizing 0.2 g tissue per 0.5 mL extraction buffer (50 mM sodium phosphate buffer pH 8.0, 150 mM NaCl, 0.2% Igepal, 5 mM EDTA, 1X complete Protease Inhibitor Cocktail solution (Roche, Basel, Switzerland)). After centrifugation at 5000 rpm for 10 min., supernatants were transferred to clean tubes and stored at –80 °C. Protein concentration was determined by the Bradford method (Bio-Rad, Hercules, CA, USA).

For immunoblot analysis, protein extracts (30 µg of protein per lane) were separated in 12% SDS–polyacrylamide gels (PAGE) and transferred to Immobilon^TM^ PDVF membranes (Millipore Co, Burlington, MA, USA). The membranes were blocked for one h in phosphate-buffered saline solution (PBS-T: 137 mM NaCl, 2.7 mM KCl, 10 mM Na_2_HPO_4_, 2 mM KH_2_PO_4,_ and 0.1% Tween-20) with 5% of non-fat dried milk at room temperature. To detect *PR-1*, the rabbit polyclonal *PR-1* antibody (Agrisera, Vännäs, Sweden, #AS10 687) was used at a 1:2500 dilution. ICS1 was detected with the rabbit polyclonal ICS1 antibody UCB68 at a 1:10,000 dilution [39]. After washing in PBS-T (three times), the membranes were incubated with the anti-mouse horseradish peroxidase-conjugated secondary antibody (KPL, #074-1806, 1:10,000) or the anti-rabbit horseradish antibody (Invitrogen, Waltham, MA, USA, #65-6120, 1:10,000). After washing in PBS-T (10 min three times), proteins immunodetected were visualized using chemiluminescence kits (Thermo Fisher Scientific, Waltham, MA, USA, #34087 and #34095) according to the manufacturer’s instructions. Chemiluminescence was detected by X-ray films, except the top right blot in Figure 1a, for which it was detected by a CCD camera (Bio-Rad ChemiDoc XRS+, Hercules, CA, USA).

### 4.5. Salicylic Acid Extraction and Quantitation

SA extracted from leaf tissues was quantified following the protocol described in Verbene et al. [59] adapted for the extraction from Arabidopsis leaves. Briefly, 0.2 to 0.3 g of tissue was collected from seedlings grown on plates or mature leaves from plants grown on soil. The collected tissue was frozen and ground in 1 ml of cold methanol (90%). Samples were homogenized with a Bioruptor^®^-Plus sonicator (Diagenode, Liege, Belgium) and centrifuged at 8000× *g*. The pellet was suspended in 500 µL of methanol 100%, and sonication and centrifugation steps were repeated. Combined supernatants were mixed with 10 mL NaOH 0.2 M and were dried using a SpeedVac (65 °C, 90 min). Then, the samples were suspended in 1 mL of TCA 5%–cyclohexane–ethyl acetate (1:2:2) and were centrifuged. The aqueous phase was rescued and combined with 800 mL cyclohexane–ethyl acetate (1:1). The organic phase was reserved. The aqueous phase was mixed with 300 μL of HCl 8 M and incubated at 80 °C for 60 min for a new organic extraction with 800 mL cyclohexane–ethyl acetate (1:1). The organic fractions obtained during the protocol were combined, mixed with 120 mL sodium acetate 0.2 M, and evaporated in a SpeedVac (45 °C, 20 min). The remainder was dissolved in 250 µL of mobile phase (water–methanol (1:1, *v*/*v*), adjusted to pH 2.0). These samples were filtered to subsequent quantitation. SA quantification was carried out by high-performance liquid chromatography equipped with a fluorescence detector (Agilent 1260 Infinity II HPLC—Agilent FLD 8 µL detector, Santa Clara, CA, USA). Analysis of SA was performed using a SunShell C18 column (150 mm × 4.60 mm i.d, pore size 2.6 µm) at 30 °C. Mobile phase was delivered through Agilent 1260 quaternary pump equipped with a degasser and autosampler using an isocratic elution. The injection volume was 20 μL, and the column flow rate was 1.0 mL/min. FLD was employed for SA detection with excitation and emission wavelengths set to 305 nm and 404 nm, respectively. The results show data from three independent experiments.

### 4.6. Genotyping

Plant DNA was extracted by cutting 5 mm^2^ of young leaves of soil-grown Arabidopsis plants. Tissue was ground in TNE/SDS buffer (200 mM TRIS, 250 mM NaCl, 2 mM EDTA, 0.5% SDS, pH 8.0). The nucleic acids were precipitated with isopropanol, washed with ethanol 70%, and resuspended in TE buffer (10 mM Tris, 1 mM EDTA, pH 8.0). DNA was used in PCR reactions using the oligonucleotides described in Supplementary Table S1.

## Data Availability

Not applicable.

## Supplementary Materials

The supporting information can be downloaded at: https://www.mdpi.com/article/10.3390/ijms231911631/s1.
