# Supplementary material for: The TGA Transcription Factors from Clade II Negatively Regulate the Salicylic Acid Accumulation in Arabidopsis
Source: Int J Mol Sci. 2022 Oct 1;23(19):11631. doi: 10.3390/ijms231911631 (PMC9569720; doi:10.3390/ijms231911631)
Supplement: Supplementary file 1 [file ijms-23-11631-s001.zip › ijms-1925790-supplementary.pdf]

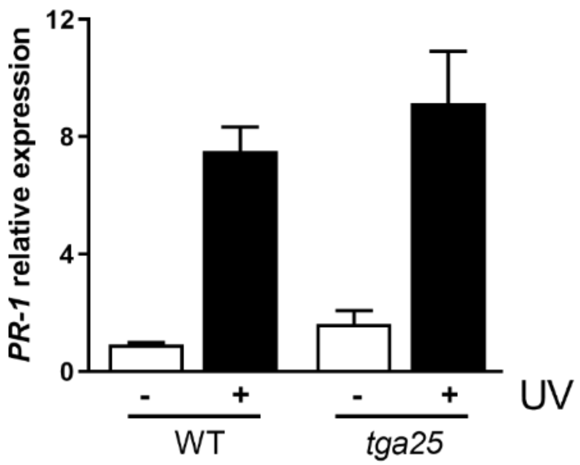

Figure S1. The PR-1 transcript is not differentially expressed in the *tga2-1*/*tga5-1* mutant plant compared to WT in response to UV-C. *PR-1* transcript was evaluated in WT and the double mutants *tga2-1*/*tga5-1* (*tga25*). 15-day-old plate-grown plants were irradiated with UV-C for 20 minutes (+). Plants covered with a UV-C filter were used as control (—). After the treatment, plants were transferred to normal growth conditions. Samples were frozen after 24 hours. RNA was extracted, cDNA was synthesized, and *PR-1* expression was evaluated by qPCR. Bars indicate the mean of the relative expression of *PR-1* ± SEM from four biological replicates (n=4). No statistical differences were detected between genotypes according to a 2-way ANOVA and Sidak's post-test (p>0.1).

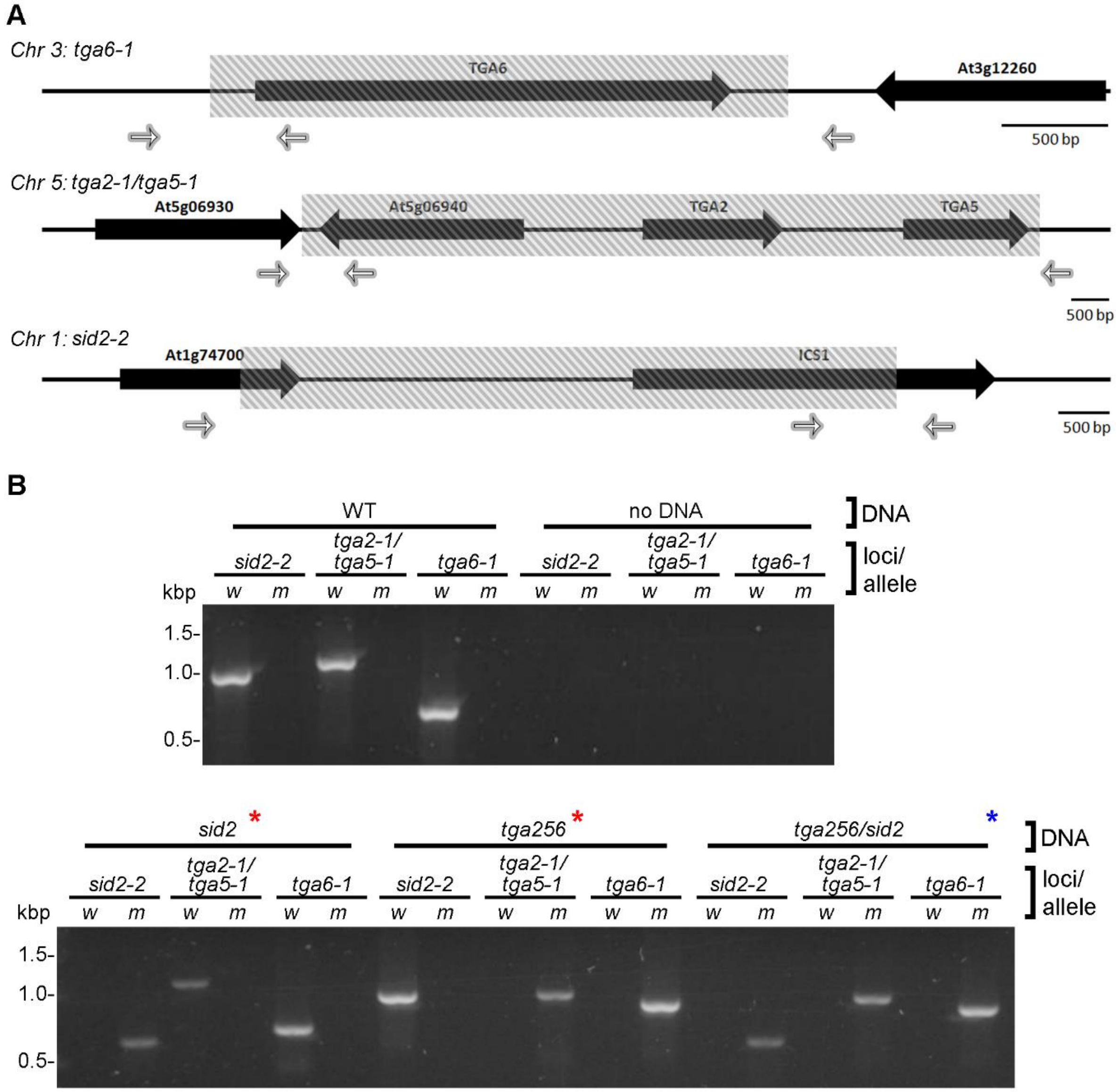

Figure S2. Identification of *tga2-1/tga5-1/tga6-1/sid2-2* quadruple mutant plant. *tga2-1/tga5-1/tga6-1* mutant plants were crossed with *sid2-2* mutant plants. According to Mendel's segregation, F2 plants were screened with a probability of 1/64 to find a quadruple mutant. Plants were screened by PCR using TNE/SDS DNA extraction and PCR with the oligonucleotides described in Table S1. **(A)** Schematic representation of deletions in the mutant plants. Black arrows represent the genes, and gray boxes indicate the deleted regions in the mutant plants. Small gray arrows represent the location of the oligonucleotides used for the PCR reactions **(B)** Genotype analysis of mutant plants. Three different loci/allele were analyzed: *sid2-2*, *tga2-1/tga5-1*, and *tga6-1*. We used two oligonucleotides mixtures to detect the wild type (*w*) or the mutant allele (*m*). The parental genotypes are indicated with red asterisks, and the quadruple mutant plant is indicated with a blue asterisk. DNA from WT plants was used as control.

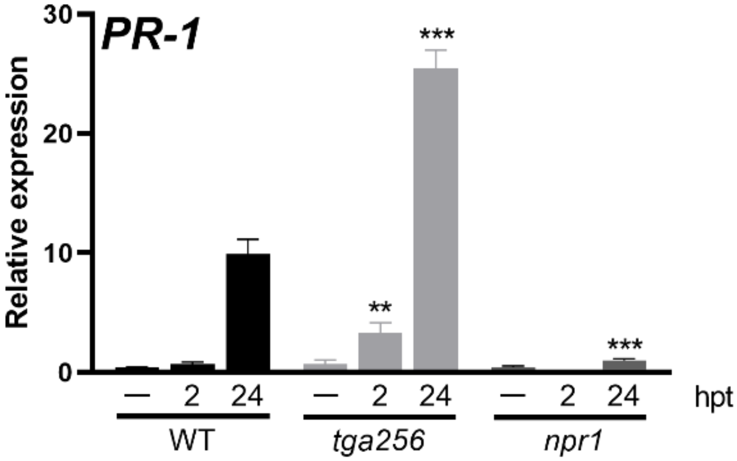

Figure S3: ***PR-1*** **expression depends on the NPR1 in UV-C treatment.** 15-day-old WT, tga2-1/*tga5-1*/*tga6-1 (tga256)*, and *npr1-2 (npr1)* mutant seedlings were treated with UV-C. Non-irradiated seedlings were used as controls (—). Treated seedlings were frozen two and 24 hour post-treatment (hpt). RNA was extracted, and the *PR-1* expression was evaluated by RT-qPCR. Bars represent the mean of the relative expression ± SEM from four biological replicates. Asterisks indicate statistically significant differences between the mutant genotypes and the WT plants according to a 2-way ANOVA analysis and Sidak's post-test (**:$p<0.01$, ***:$p<0.001$).

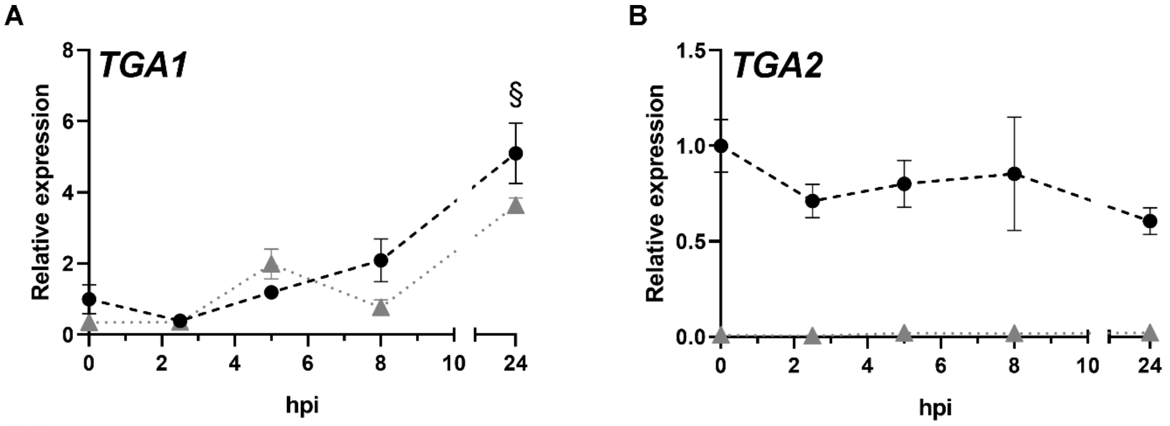

Figure S4: The TGA2 gene is not induced by the treatment that induces the SA accumulation. 4-week-old WT (black circles) and *tga256* mutant (gray triangles) plants were syringe-infiltrated with Pst DC3000/AvrRPM1 ($OD_{\lambda 600}$=0.01). Samples were collected 2.5, 5, 8, and 24 hours post inoculation (hpi). Noninoculated plants were used as controls (0 hpi). RNA was extracted, and the gene expression of **(A)** *TGA1* and **(B)** *TGA2* was evaluated by RT-qPCR. The data represents the mean of the relative gene expression ± SEM from 4 biological replicates. No differences were detected in the *TGA1* expression between genotypes. § indicate a statistically significant difference compared to the control condition and time zero. No statistically significant differences were detected in the *TGA2* expression in WT plants compared to time zero (p>0.05). As expected, *TGA2* was not detected in the *tga256* genotype.

Table S1. Oligonucleotides

	AGI	Gene		Sequence
RTqPCR	AT2G14610	*PR-1*	Fw	ACACGTGCAATGGAGTTTGTGG
			Rv	TTGGCACATCCGAGTCTCACTG
	AT3G48090	*EDS1*	Fw	CGAAGGGGACATAGATTGGA
			Rv	ATGTACGGCCCTGTGTCTTC
	AT3G52430	*PAD4*	Fw	GCAAGTATCTTGCGTTGTGC
			Rv	TAAAGACTGGCGGGCATTAC
	AT5G26920	*CBP60g*	Fw	AATAACGAGGAGGATGAGAACG
			Rv	TCAGACACGGTAAGAAACATCG
	AT1G73805	*SARD1*	Fw	CCTCAACCAGCCCTACGTTA
			Rv	TAGTGGCTCGCAGCATATTG
	AT4G39030	*EDS5*	Fw	GTGACAAGAAGTGGCTATGGTTT
			Rv	GACTCGGCCCATCTGAATTA
	AT5G13320	*PBS3*	Fw	GCGTTGTTGTAGAAACCAGTCACC
			Rv	GTTGTCACAAATTTCGCTGGCTTG
	AT5G65210	*TGA1*	Fw	ACGAACCTGTCCATCAATTCGG
			Rv	CCATGGGAAGTATCCTCTGACACG
	AT5G06950	*TGA2*	Fw	AAAGCTTCTGGCGAATCAGTTGG
			Rv	TGACTGTTGTAAGCTCTCCATCCC
	AT5G08290	*YLS8*	Fw	TTACTGTTTCGGTTGTTCTCCATTT
			Rv	CACTGAATCATGTTCGAAGCAAGT
	AT1G74710	*ICS1*	Fw	CACTGCAGACACCTAATTGAGTCC
			Rv	GCTTGGCTAGCACAGTTACAGC
Genotype analysis	AT1G74710	*sid2-2*	Fw Mut	TTCTTCATGCAGGGGAGGAG
			Fw WT	CAACCACCTGGTGCACCAGC
			Rv	AAGCAAAATGTTTGAGTCAGCA
	AT5G06950/AT5G06960	*tga2-1/tga5-1*	Fw	CTTTCCTCGGCAAGTCAATC
			Rv WT	CCCAAGCTCTCTGATTTTGC
			Rv mut	TATGTTGTGACCGGACCAGA
	AT3G12250	*tga6-1*	Fw	CAGCAACCAAATTCCATCG
			Rv WT	CAAGCCTCCCAGGAGTGA
			Rv mut	AGCAGCGTCACCACATCA
